# Detection of Q129H Immune Escape Mutation in Apparently Healthy Hepatitis B Virus Carriers in Southwestern Nigeria

**DOI:** 10.3390/v13071273

**Published:** 2021-06-29

**Authors:** Olufisayo Adeyemi Adesina, Olusola Anuoluwapo Akanbi, Oluyinka Oladele Opaleye, Margaret Oluwatoyin Japhet, Bo Wang, Adekemi Olubukunola Oluyege, Patrycja Klink, C.-Thomas Bock

**Affiliations:** 1 Department of Microbiology, Obafemi Awolowo University, Ile-Ife 220282, Nigeria; adesinafat@yahoo.co.uk (O.A.A.); megdeoti@yahoo.com (M.O.J.); 2 Department of Microbiology, Ekiti State University, Ado-Ekiti 360213, Nigeria; adekemi.oluyege@eksu.edu.ng; 3 Division of Viral Gastroenteritis and Hepatitis Pathogens and Enteroviruses, Department of Infectious Diseases, Robert Koch Institute, 13353 Berlin, Germany; olusola.akanbi@ncdc.gov.ng (O.A.A.); bowang@vt.edu (B.W.); klinkp@rki.de (P.K.); 4 Department of Medical Microbiology and Parasitology, Ladoke Akintola University of Technology, Ogbomoso 230232, Nigeria; yopaleye@yahoo.com; 5 Institute of Tropical Medicine, University of Tuebingen, 72076 Tuebingen, Germany

**Keywords:** hepatitis, mutation, resistance, PCR, phylogeny, Nigeria

## Abstract

As the global effort to eradicate hepatitis B continues, immune escape mutations (IEMs) and drug resistance mutations (DRMs) affecting its diagnosis, treatment, and prevention are compromising this goal. However, knowledge about the prevalence and circulation of these mutations in Nigeria is scarce. Serum samples (*n* = 199) from apparently healthy prospective blood donors, pregnant women, and individuals presenting with fever in southwestern Nigeria were analyzed for the presence of IEMs and DRMs by means of nested PCR in the HBV S (HBs) and HBV polymerase (Pol) genes, followed by phylogenetic and mutational analyses. In total, 25.1% (*n* = 50/199) of samples were positive for HBV, as measured by PCR. In 41 samples (20.6%), both fragments could be amplified, whereas the HBs gene and the Pol gene fragment alone were detected in 0.5% (*n* = 1/199) and 4% (*n* = 8/199) of samples, respectively. Sequences were successfully obtained for all 42 HBs gene fragments but for only 31/49 Pol gene fragments (totaling 73 sequences from 44 individuals). All sequences were identified as HBV genotype E. IEMs were present in 18.2% (*n* = 8/44) of the sequences of HBV-positive individuals with available sequences. IEM Q129H was detected in eight out of the 44 (18.2%) HBV isolates sequenced in this study; however, no DRMs were observed. This study confirms the circulation of HBV IEMs and reports the presence of Q129H IEM for the first time in Nigeria. Intensified research on the dynamics of IEM is necessary in order to enhance the elimination of HBV.

## 1. Introduction

Infections with the hepatitis B virus (HBV) are a major global health problem, leading to acute and chronic liver disease. According to the World Health Organization (WHO) in 2015, 257 million people were chronic HBV carriers, resulting in about 887,000 deaths per year, mainly from complications such as cirrhosis and hepatocellular carcinoma (HCC). However, out of the high number of infected individuals, only 27 million are aware of their infection [1]. HBV is a member of the *Hepadnaviridae* family, genus *Orthohepadnavirus*. Its partially double-stranded DNA consists of four overlapping open reading frames (ORFs), encoding for the surface proteins (PreS1/PreS2/S), the polymerase (Pol), the capsid proteins (PreC/C), and the X protein. Unlike other DNA viruses, HBV has a high mutation rate due to its replication via an intermediate RNA by means of a reverse transcriptase lacking proofreading ability [2,3].

Mutations in the HBV genome have been observed across all four ORFs in both acute and chronic HBV-infected individuals [4]. Some of the HBV mutations, caused by the host’s immune pressure and external pressure such as vaccination or antiviral therapy, have been reported to favor viral fitness and hence viral replication. These mutations are termed immune escape mutations (IEMs) and are found in the “*a*” determinant on the major hydrophilic region (MHR) of the HBV surface antigen (HBsAg), which is a region containing a cluster of B-cell epitopes which are crucial for the binding to neutralizing-antibodies. Due to the overlap of the S and reverse transcriptase (RT) gene, drug resistance mutations (DRMs) on the RT can lead to the emergence of IEMs in the MHR and vice versa [5,6]. It has been reported that mutations which escape neutralizing antibodies enhance persistent infection, promote viral fitness, and can lead to the reactivation of HBV infection during weak or suppressed immune responses [7,8,9]. Furthermore, the escape from or evasion of neutralizing antibodies (from hepatitis B immunoglobulin and HBV vaccine) gives rise to false negative results with the use of diagnostic kits and/or an underestimation of HBsAg levels [9,10,11].

The first described vaccine escape mutation, arising from the substitution of glycine (G) with arginine (R) at position 145 (G145R) within the *a-*determinant region of the S gene, was reported in the sera of Italian vaccinated children who had HBsAg and anti-HBs antibodies [12]. In the following years, other mutations observed on the *a-*determinant, which are considered as immune escape variants, including T116N, P120S/E, I/T126A/N/I/S, Q129H/R, M133L, K141E, P142S, and D144A/E, have also been reported [13]. Furthermore, the IEMs Q129N, M133T, and F134Y have been predicted to cause HBV diagnostic failure [14].

Genetic changes, such as substitutions, deletions, and recombination in the PreS/S ORF, can emerge as a result of various factors, such as the infidelity of the polymerase, viral fitness, the strict relationship of the products of the S and Pol genes, and the immune system of the host, as well as external pressure due to vaccination or antiviral therapy [2,5]. Since early 1980s, vaccines have been administered to prevent HBV infection; however, changes in the S gene may arise and lead to failed immunoprophylaxis and the creation of escape mutants [15].

Nigeria has long been among the most highly HBV endemic countries in sub-Saharan Africa, with an estimated prevalence of between 2.5% and 40%, depending on the studied populations within the entire nation [16,17]. A systematic review on the prevalence of HBV in Nigeria covering the years 2000–2013 reported a prevalence range of 0.5% to 46.8% among the published literature [18]. Nevertheless, there is lack of robust epidemiological data, limited awareness, and an immense need for proper diagnosis and clinical care regarding HBV infection in Nigeria, especially regarding IEMs and DRMs.

## 2. Materials and Methods

### 2.1. Sampling

In total, 199 serum samples were obtained from the venous blood samples collected from pregnant women (*n* = 97), sick individuals presenting with fever and other mild conditions (such as headache and gastrointestinal disturbance) (*n* = 30), and apparently healthy prospective blood donors (*n* = 72). Samples were collected in selected primary and secondary medical facilities and communities from Ekiti, Lagos, Ondo, Osun, and Oyo States, in southwestern Nigeria, between October 2016 and February 2018. All samples in this study were randomly selected from a pool of 787 blood samples collected for hepatitis E virus studies. A structured questionnaire was administered to gather socio-demographic information, health status, and travel history from each consenting subject involved in the study.

### 2.2. Ethical Consideration

Ethical approval (Protocol number—OSHREC/PRS/569T/3) was obtained on 30th November 2016 from the Medical Advisory Committee of Seventh-Day Adventist Hospital, Ile-Ife, Nigeria, and appropriate permissions were obtained from relevant authorities in the facilities used and informed consent was received from every participant before sample collection. The confidentiality right of the subjects was maintained as their identities were made anonymous and the data obtained were coded as appropriate.

### 2.3. Viral Nucleic Acid Extraction

Viral nucleic acids were extracted from 135 µL of each serum sample with the Qiacube BioRobot Workstation (QIAgen, Hilden, Germany) using the QIAamp MinElute Virus Spin Kit (QIAgen, Hilden, Germany) according to the manufacturer’s instructions. RNA was eluted in 60 µL and stored at −80 °C until further use.

### 2.4. Amplification and Sanger Sequencing

Real-time PCR for the quantification of HBV DNA was carried out using virus-specific primers and a Taqman probe targeting a 90-bp-long fragment in the conserved region of the HBs gene of the HBV genome (position 182–271 based on GenBank number X75657) using the Superscript III/Platinum one step RT-PCR kit (Invitrogen/Thermo Fisher Scientific, Planegg, Germany) on a LightCycler 480 (Roche Diagnostics Corporation, Rotkreuz, Switzerland). The real-time PCR was performed in a volume of 25 µL, consisting of 0.75 µL each of 10 µM forward primer HBV-61 GGA CCC CTG CTC GTG TTA CA and reverse primer HBV-62 GAG AGA AGT CCA CCA CGA GTC TAG A, 4 µL qPCR Mix 5X, 0.25 µL Probe HBV TM-05 (FAM- TGT TGA CAA RAA TCC TCA CAA TAC CRC AGA -DB), 9.25 µL PCR grade water, and 5 µL of the template. The cycling condition was an initial hold of 2 min at 50 °C,10 min at 95 °C, followed by 45 cycles of 95 °C for 10 s and 60 °C for 34 s.

For genotyping, primer sets covering the highly conserved overlapping regions of the S and Pol gene of the HBV genome were amplified. Amplification of the HBs gene (First product 409 bp, location 414–822 and second product 341 bp, location 455–795, based on GenBank number X75657) was carried out by means of nested PCR with previously described primer sets and PCR conditions [19] using the HotStarTaq Master Mix Kit (QIAGEN, Hilden, Germany) and a Biometra cycler. Briefly, the mix for the first round HBs gene PCR was a 12.5 µL reaction consisting of 0.5 µL each of primers HBV-22 (forward) TGC TGC TAT GCC TCA TCT TC and reverse primers HBV-065 (CAA AGA CAA AAG AAA ATT GG) and HBV-066 (CAC AGA TAA CAA AAA ATT GG), 6.25 µL of HotStar mix, 1 µL of the template, and 3.75 µL of PCR grade water. The thermal cycling conditions were an initial denaturation of 15 min at 95 °C, followed by 35 cycles of 30 s at 94 °C denaturation; 30 s at 55 °C annealing; 30 s at 72 °C extension, and a final extension for 10 min at 72 °C. The second round of PCR consisted of 0.25 µL each of primers HBV-024 (forward primer—CAA GGT ATG TTG CCC GTT TGT CCT) and reverse primers HBV-041 GGA CTC AMG ATG YTG TAC AG and HBV-064 GGA CTC AMG ATG YTG CAC AG., 6.25 µL of HotStarTaq mix, 1 µL of the template, and 4.5 µL of PCR-grade water. The cycling conditions were the initial denaturation of 15 min at 95 °C, followed by 30 cycles of 30 s at 94 °C denaturation, 30 s at 50 °C annealing, 30 s at 72 °C extension, and a final extension for 5 min at 72 °C. The Pol gene was amplified by means of nested PCR (First product size 1228 bp, location 47–1274; second round product 1143 bp, location 57–1199 based on GenBank number X75657) using the HotStarTaq Master Mix Kit (QIAGEN, Hilden, Germany) and a Biometra cycler. Briefly, the mix for the first round Pol-PCR was a 12.5 µL reaction consisting of 0.5 µL each of forward primer HBV-18 CTG TAT CTT CCT GCT GGT GGC T and reverse primer HBV-16 GCA GTA TGG ATC GGC AGA GGA, 6.25 µl of HotStar mix, 1 µL of the template, and 4.25 µL of PCR-grade water. The thermal cycling conditions were an initial denaturation of 15 min at 95 °C, followed by 35 cycles of 30 s at 94 °C denaturation; 30 s at 56 °C annealing; 90 s at 72 °C extension, and a final extension for 10 min at 72 °C. The second round of PCR consisted of 0.25 µL each of forward primer HBV-1 CTG CTG GTG GCT CCA GTT CAG GA and reverse primer HBV-17 GGG GTT GCG TCA GCA AAC ACT, 6.25 µL of HotStar mix, 1 µL of the template, and 4.75 µL of PCR-grade water. The cycling conditions were the same except that the final extension time was 7 minutes and the number of cycles was 40. PCR products were visualized on a 1.5% agarose gel stained with GelRed (Biotium^®^, Fremont, CA, USA) in 1% TAE.

Samples with a band at the expected nested size of 341 bp and 1143 bp, respectively, were subjected to cleaning with ExoSAP-IT™ reagent (Applied biosystems/Thermo Fischer Scientific, Planegg, Germany), according to the manufacturer’s instructions. Sanger sequencing was done for the S gene fragments using forward primer HBV-24 CAAGGTATGTTGCCCGTTTGTCCT, 0.5 µL BigDye version 3.1 (Applied Biosystems/Thermo Fisher Scientific, Planegg, Germany), 1.5 µL buffer, and 1 µL of the cleaned PCR product in an automated DNA sequencer ABI 3130xl (Applied Biosystems/Thermo Fisher Scientific, Planegg, Germany). Sequencing PCR conditions consisted of one cycle of 96 °C for 1 min; 25 cycles of 96 °C for 30 s and 45 °C for 15 s; and then 60 °C for 4 mins. The same conditions and protocols were used to sequence the Pol gene using forward primers HBV-1 CTG CTG GTG GCT CCA GTT CAG GA and HBV-9 GCC CGT TTG TCC TCT AAT TCC A and reverse primers HBV-10 AGC GGT ATA AAG GGA CTC ACG and HBV-17 GGG GTT GCG TCA GCA AAC ACT.

### 2.5. HBsAg Screening

After performing PCR, HBsAg screening was carried out on all PCR-positive samples using the Wantai HBsAg ELISA kit (Beijing Wantai Biological Pharmacy, China). The procedure was carried out following the manufacturer’s instructions.

### 2.6. Phylogenetic and Mutation Analyses

The HBs and Pol gene sequences generated in this study were aligned with sequences of HBV genotypes A–J and further randomly selected HBV sequences from Nigeria and other African countries, downloaded from GenBank. Additionally, for each sequence the best BLASTn [20] match (according to the highest Max score) was included in the phylogenetic analysis. An HBV sequence from a chimpanzee (Accession number D00220) was used as the outgroup. Alignments were generated using the MAFFT algorithm with default settings in Geneious Prime software version 2020.2.3 (https://www.geneious.com; accessed on 20th August 2018). Maximum likelihood phylogenetic trees with 1000 bootstrap replicates were constructed using IQ-TREE v1.6.12 [21] after choosing the best fitting model determined by the ModelFinder implemented in IQ-TREE v1.6.12 [22]. The trees were graphically adjusted using iTOL [23].

Sequences from the Pol gene ORF were examined for DRMs using BioEdit version 7.2.6 [24] and geno2pheno software (https://hbv.geno2pheno.org/; accessed on 20th August 2018. The S and Pol gene nucleotide sequences from this study were submitted to the GenBank Nucleotide Sequence Database under the accession numbers MN819022—MN819064, MZ368887, MZ368888, and MZ398363—MZ398367.

### 2.7. Data Analysis

The Chi-squared test was performed to test for significant correlations between the different factors, using SPSS version 21 (IBM Corp. Released 2012. IBM SPSS Statistics for Windows, Version 21.0. Armonk, NY, USA).

## 3. Results

### 3.1. Characteristics of the Study Cohort

In this study, 199 serum samples from pregnant women (*n*= 97), apparently healthy prospective blood donors (*n*= 72), and individuals presenting with fever (*n*= 30) were analyzed for the presence of HBV. The study cohort included mainly females (77.9%; *n*= 155/199) and the dominant age group was 21–30 years (38.7%, *n* = 77/199), closely followed by 31–40-year-olds (37.2%; *n* = 74/199) (Table 1). None of the study participants presented with hepatitis or liver-related problems and none was known to be on treatment of any condition related to cancer, hepatitis, or any liver-related disease based on the data gathered from the administered questionnaire.

### 3.2. HBs and Pol Gene Specific PCR

In total, HBV (HBs gene- and/or Pol gene-positive PCR) was detected in 25.1% (*n* = 50/199) of individuals. Of these 50, 20%, (*n* = 10) were from Ekiti, 26% (*n* = 13) from Lagos, 10% (*n* = 5) from Ondo, 28% (*n* = 14) from Osun, and 16% (*n* = 8) from Oyo States. Those who were positive for both HBs and Pol genes comprised 20.6% (*n* = 41/199) of the sample, whereas the HBs gene only was detected in 0.5% (*n* = 1/199) and the Pol gene only was detected in 4% (*n* = 8/199) (Table 2). The HBs gene was detected in 21.1% (*n*=42/199; 10 from Ekiti, 13 from Lagos, 0 from Ondo, 11 from Osun, and 8 from Oyo States). Among the 42 HBs-PCR-positive samples, 50% (*n*=21) were from pregnant women, 35.7% (*n*=15) from apparently healthy and 14.3% (*n*=6) from sick individuals presenting with fever and other mild conditions. In all, 81% (*n*=34) and 19% (*n*=8) of the HBs-PCR-positive samples were from female and male individuals, respectively. On the other hand, the HBV Pol gene was detected in 24.6% (*n*=49/199) of the analyzed samples, out of which 10, 13, 5, 14 and 7 were from Ekiti, Lagos, Ondo, Osun, and Oyo States, respectively. Out of the 49 Pol gene PCR-positive samples, 81.6% (*n*=40) and 18.4% (*n*=9) were from female and male individuals, respectively, whereas 55.1% (*n* = 27) were pregnant women, 30.6% (*n* = 15) apparently healthy, and 14.3 % (*n* = 7) individuals presenting with fever, respectively.

No statistically significant correlation was observed with the sex and categories of the study participants.

### 3.3. HBsAg ELISA

All the 50 samples that were HBV PCR-positive were also HBsAg-positive. No occult HBV infections were observed in the study.

### 3.4. Phylogenetic and Mutation Analyses

**Figure 1:**
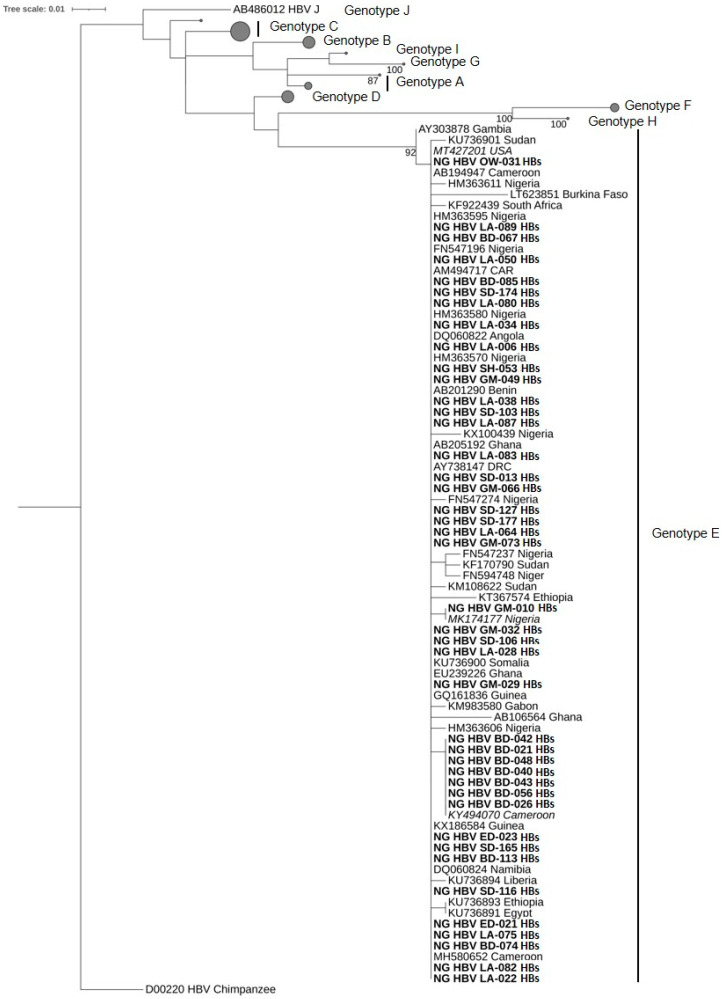
Phylogenetic analysis constructed from a 272-bp-long fragment of the HBs gene of HBV using the maximum likelihood method based on the TVM+I+G4 model. All 42 sequences clustered within genotype E. The HBV sequences from this study are indicated in bold characters. The best BLASTn matches are presented in italics. HBV reference genomes of genotypes A–D and F–J are collapsed with the size of the gray circles being proportional to the number of reference sequences. Bootstrap values >75% are indicated.

**Figure 2:**
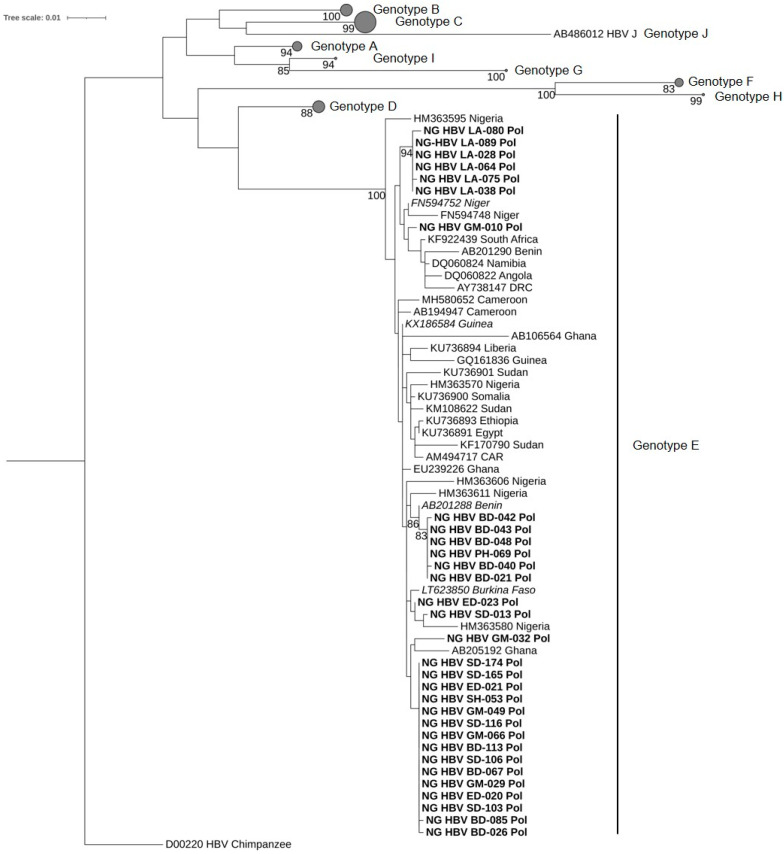
Phylogenetic analysis inferred using the maximum likelihood method based on the TIMe-+I+G4 model reconstructed from a 1014-bp-long fragment of the partial HBV Pol gene region. All 31 sequences obtained from this study were assessed as genotype E. The HBV sequences from this study are indicated in bold, whereas the best BLASTn matches are indicated in italics. HBV reference genomes of genotypes A–D and F–J are collapsed, with the size of the gray circles being proportional to the number of reference sequences. Bootstrap values >75% are indicated.

Of 42 and 49 samples positive in the HBs and the Pol gene PCR, respectively, 42 (100%) and 31 (63.5%) were successfully sequenced, corresponding to 73 sequences from 44 isolates. The sequences of the partial HBs gene fragment share 99.2%–100% identity to each other, whereas the sequences of the partial Pol gene fragment show an identity between 98.2%–100% to each other. Available Nigerian as well as further African HBV-sequences, together with reference sequences representing HBV genotypes A–J, were randomly selected from GenBank and used for phylogenetic analysis. The phylogenetic trees were constructed via the maximum likelihood method based on the TVM+I+G4 model (Transversion model, AG=CT and unequal base frequency, invariable sites, and discrete gamma distribution with four categories) for the Pol gene sequences and the TIMe+I+G4 model (Transition model, AC=GT, AT=CG and equal base frequency, invariable sites, and discrete gamma distribution with four categories) for the HBs gene sequences using IQ-TREE v1.6.12. All 73 sequences obtained in this study were classified as HBV genotype E (Figure 1 and Figure 2).

#### Immune Escape Mutation

**Figure 3:**
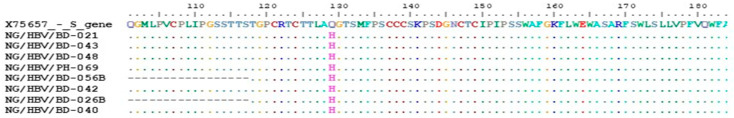
Partial HBs and Pol gene sequences obtained from this study aligned with the HBV genotype E reference sequence (Accession number X75657), showing the escape mutation at amino acid 129 of the S gene (sQ129H).

The mutational analysis of the partial HBs and Pol gene sequences revealed the presence of the escape mutation Q129H in eight HBV isolates sequenced in this study (Figure 3). In total, the sQ129H IEM was detected in 18.2% (*n* = 8/44) of the individuals with available sequence. Where available, the longer Pol gene sequence was submitted, otherwise the HBs gene sequence was used. The sequences with accession numbers MZ398363 (BD-021), MZ398364 (BD-042), MZ398365 (BD-043), MZ398366 (BD-040) and MZ398367 (BD-048) MZ368887 (BD-026B), MZ368888 (BD-056B), and MN819056 (PH-069) have the sQ129H mutation on the HBs part. Statistical analysis showed a correlation of the observed IEMs with sample location (*p* = 0.00) but not the categories of study participants (*p* = 0.06).

Based on the information gathered from the administered questionnaire, none of the subjects with IEMs in this study was on any hepatitis medication. Further details about each of the individuals is given in Table 3.

Out of the 31 Pol gene sequences obtained, a predominance of the M336L mutation (96.8%; *n* = 30) was observed. Other mutations detected on the Pol gene sequences were N248H (2.6%; *n* = 7); E263D (19.4%; *n* = 6); K11R (6.5%; *n* = 2); S53I (19.4%; *n* = 6); and one (3.2%) each of L77V, D134H, V56M, and D271N mutations, none of which has been reported in HBV genotype E, and the significance of this is not yet known.

## 4. Discussion

Escape mutations in general have been reported to be responsible for vaccine- and diagnostic-escape phenomena. When found in immune-compromised individuals, HBV reactivation is defined by Terrault et al. [25] as the abrupt reappearance of HBV (serum HBV DNA > 100 IU/mL) in the serum of a person with previously resolved infection or a marked increase of HBV replication (>2 log increase of serum HBV DNA from baseline level) in an immunosuppressed patient with previously stable chronic infection, and can ensue in formerly anti-HBs-positive individuals [2]. Studies have shown that escape mutations and their consequences have been found in both vertically transmitted and newly acquired mutations in previously vaccinated persons [2,13,26].

In this study, the sQ129H mutation was detected in sequences of 18.2% (*n* = 8/44) of HBV-positive individuals, which is the highest IEM prevalence reported so far in Nigeria. The IEMs were detected in the HBV sequences of samples from six apparently healthy prospective blood donors in Ekiti and two pregnant women each from Ekiti and Ondo States, Nigeria. All sequences obtained in this study were classified as HBV genotype E, which is known to show a clear genotypic divergence from all genotypes within the a determinant, where escape mutations can occur [27,28], reported that the types of polymorphisms at positions associated with escape mutations observed in HBV vary from one genotype to another and that the most common of these polymorphisms found in genotype E are T116N, P120L/S, Q129H/R, M133I, D144E, and G145I. Different studies report the presence of the Q129H mutation in different HBV genotypes, including genotype E [29,30,31], whereas the escape mutation Q129R has been detected in genotypes A, C, and D and Q129L in genotype F only.

In previous studies performed in Nigeria, IEMs at various positions were detected in different populations. Faleye et al. [16] performed two studies in Ibadan, Southwest Nigeria, analyzing pregnant women and apparently healthy community dwellers, and reported the occurrence of a specific pattern of IEMs in both studied groups. As this pattern has not been reported in other Nigerian communities to date, they conclude that this strain might be a recent development. In both studies other IEMs were also detected; however, these did not include the IEM sQ129H, which was detected in this study. The difference in the observed mutations may not be clearly explained but could be due to host, viral, or environmental factors. However, the results imply that different HBV-strains harboring IEMs are in circulation in southwestern Nigeria, thus calling for more intensive studies on different categories of people in the area.

Apart from corroborating the occurrence of IEMs in Nigeria, this study reports for the first time the detection of the Q129H mutation from samples collected in Ekiti and Ondo States, southwestern Nigeria.

In southwestern Nigeria, certain common practices that predispose a greater percentage of the population to HBV infection include local uvulectomy, scarification, sharing of sharps, inscription of tribal marks, traditional circumcision, blood oaths, dental procedures, local hair shaving, manicure/pedicure, and delivery of babies at home. These practices, as mentioned by [17,32], sustain the circulation of HBV in the region and also create a greater chance for the selection of escape and various other mutations. The presence of sQ129H in Africa has so far only been reported from HBV-HIV-coinfected patients on antiretroviral therapy (ART) in Cote D’Ivoire, Ethiopia, and Angola [15,30,33,34]. Although the HIV status of the participants in this study was not determined to further confirm the relationship between HIV infection/ART and IEM occurrence, it is an important aspect to investigate in order to gain better insights. An understanding of the extent to which IEMs circulate in Africa, and Nigeria in particular, is essential to improving HBV diagnosis and therapy in patients with and without HIV coinfection.

Although the existence and circulation of escape mutants was already reported in 1990, the first case reported in Nigeria was the G145K/R escape mutation, detected in an asymptomatic pregnant woman in Ibadan, Oyo State, among samples collected in 2012 [35].

The sG145R mutation is one of the most widely reported escape mutations and its horizontal transmission has been proven frequently [36,37,38,39]. There is no information about the transmissibility of sQ129H/L/R/N until today. However, the detection of Q129H among pregnant women and prospective blood donors detected in this study calls for more studies on its transmissibility, other likely effects on its carriers, as well as its interaction with the existing HBV vaccine, in order to prevent its further spread. In Botswana, Choga et al. [40] reported a prevalence of escape mutations of 14% from HBV isolates of blood donors and 15% from isolates of HBV/HIV-co-infected patients. In a study on Pakistani blood donors, Harris et al. [41], identified IEMs in 14% of HBsAg-reactive donors and reported that the donors harboring the escape mutations were a largely unvaccinated population. These findings, together with the results from our study, emphasize the risk of blood-borne virus transmission through improperly screened blood for donation or transfusion. The need for more detailed epidemiologic studies on the success or failure of HBV vaccination programs in Africa cannot be overemphasized in order to understand the correlation between the frequency of escape mutations and genotypes, as well as the impact of escape mutations in different genotype backgrounds on the performance of commercially available HBsAg assays.

None of the eight study participants carrying the sQ129H escape mutation in this study had blood transfusion records and only two, a male and a pregnant woman, claimed to have received an HBV vaccination. This suggests that the escape mutation might be caused by selection due to host/viral factors without the influence of vaccination or anti-HBV immunoglobulin therapy, as opined by [42,43].

Although an effective preventive measure through an extended HBV immunization program, leading to a dramatic reduction in the incidence of hepatitis B, is being carried out in many countries, including Nigeria, globally HBV infection still represents a major public health problem. The divergence within the a-determinant of HBV genotype E from all other genotypes is posing a challenge to the effectiveness of the current hepatitis B vaccine to efficiently protect against HBV infection as the vaccine has the HBsAg genotype A2. A similar challenge faces the vaccine in Central and South America and some Asian countries, such as Japan and Korea, where HBV genotypes F and C are predominant, respectively [44,45,46]. To further question the role of the current HBV vaccine in escape mutation, Hsu et al. [47] found that vaccinated subjects had a tendency towards higher mutation rates in a determinant of HBsAg than unvaccinated subjects.

A limitation of this study is that laboratory parameters such as viral load, liver transaminases, as well as anti-HBS titers, were not analyzed. The analyzed samples were randomly selected from a pool of 787 blood samples originally collected for hepatitis E virus studies. Further, the study group mainly comprised apparently healthy individuals, including outpatients, blood donors, and pregnant women, so the determination of these parameters was not performed at the time of sample collection.

## 5. Conclusions

The findings in this study indicate an ongoing unnoticed circulation of HBV with IEMs in southwestern Nigeria. The high prevalence of the sQ129H escape mutation detected here points out the need for better screening procedures for HBV-infected individuals in order to avoid the spread of viral strains evading vaccine protection and thus posing a serious threat to the successful elimination of HBV.

## Figures and Tables

**Table 1:** Overview of the characteristics of the cohort.

	States of Sample Collection N (%)					
	Ekiti	Lagos	Ondo	Osun	Oyo	Total
**Total**	20(10.1)	34(17.1)	28(14.1)	86(43.2)	31(15.6)	199
**Sex**						
Female	12(6.0)	33(16.6)	28(14.1)	57(28.6)	25(12.6)	155(77.9)
Male	8(4.0)	1(0.5)	0(0)	29(14.6)	6(3.0)	44(22.1)
**Study categories**						
Pregnant	5(2.5)	32(16.1)	27(13.6)	22(11.1)	11(5.5)	97(48.7)
Apparently healthy	14(7.0)	2(1.0)	0(0)	43(21.6)	13(6.5)	72(36.2)
Sick	1(0.5)	0(0)	1(0.5)	21(10.6)	7(3.5)	30(15.1)
**Age group**						
11–20	0(0)	2(1)	1(0.5)	4(2.0)	6(3.0)	13(6.5)
21–30	13(6.5)	14(7.0)	15(7.5)	25(12.6)	10(5.0)	77(38.7)
31–40	5(2.5)	17(8.5)	12(6.0)	32(16.1)	8(4.0)	74(37.2)
41–50	2(1)	1(0.5)	0(0)	16(8.0)	4(2.9)	23(11.6)
51–60	0(0)	0(0)	0(0)	4(2.0)	1(0.5)	5(2.5)
>60	0(0)	0(0)	0(0)	5(2.5)	2(1)	7(3.5)

**Table 2:** Distribution of HBV-PCR-positive samples among the cohort in the States.

States of Sample Collection N(%)						
	Ekiti	Lagos	Ondo	Osun	Oyo	Total
**Pregnant**	**5**	**32**	**27**	**22**	**11**	**97**
HBs and Pol positive	3(3.1)	13(13.4)	0(0)	3(3.1)	1(1)	20(20.6)
Pol only positive	0(0)	0(0)	5(5.2)	2(2.1)	0(0)	7(7.2)
HBs only positive	0(0)	0(0)	0(0)	0(0)	1(3.1)	1(49.5)
Total HBV positive	3(3.1)	13(13.4)	5(5.2)	5(5.2)	2(2.1)	28(28.9)
**Apparently healthy**	**14**	**2**	**0**	**43**	**13**	**72**
HBs and Pol positive	7(9.7)	0(0)	0(0)	5(6.9)	3(4.2)	15(20.8)
Pol only positive	0(0)	0(0)	0(0)	0(0)	0(0)	0(0)
HBs only positive	0(0)	0(0)	0(0)	0(0)	0(0)	0(0)
Total HBV positive	7(9.7)	0(0)	0(0)	5(6.9)	3(4.2)	15(20.8)
**Sick**	**1**	**0**	**1**	**21**	**7**	**30**
HBs and Pol positive	0(0)	0(0)	0(0)	3(10)	3(10)	6(20)
Pol only positive	0(0)	0(0)	0(0)	1(3.3)	0(0)	1(3.3)
HBs only positive	0(0)	0(0)	0(0)	0(0)	0(0)	0(0)
Total HBV positive	0(0)	0(0)	0(0)	4(13.3)	3(10)	7(23.3)
**Total cohort**	**20**	**34**	**28**	**86**	**31**	**199**
Total HBV positive	10(5)	13(6.5)	5(2.5)	14(7)	8(4)	50(25.1)

**Table 3:** Characteristics of subjects with immune escape mutations.

Sample Code	State	Sex	Category	HBV Vaccination Status	Blood Transfusion History
BD-021	Ekiti	Male	Apparently healthy	vaccinated	No
BD-026B	Ekiti	Female	Apparently healthy	unvaccinated	No
BD-040	Ekiti	Female	Apparently healthy	unvaccinated	No
BD-042	Ekiti	Female	Pregnant	vaccinated	No
BD-043	Ekiti	Female	Apparently healthy	unvaccinated	No
BD-048	Ekiti	Male	Apparently healthy	unvaccinated	No
BD-056B	Ekiti	Female	Apparently healthy	unvaccinated	No
PH/-069	Ondo	Female	Pregnant	unvaccinated	No

## Data Availability

The gene sequences obtained in this study have been submitted to the GenBank Nucleotide Sequence Database under the accession numbers MN819022—MN819064, MZ368887, MZ368888 and MZ398363—MZ398367; whereas other data are contained in this article.
